# A Passive RF Testbed for Human Posture Classification in FM Radio Bands

**DOI:** 10.3390/s23239563

**Published:** 2023-12-01

**Authors:** João Pereira, Eugene Casmin, Rodolfo Oliveira

**Affiliations:** 1 Departamento de Engenharia Electrotécnica e de Computadores, Faculdade de Ciências e Tecnologia (FCT), Universidade Nova de Lisboa, 2829-516 Caparica, Portugal; jt.pereira@campus.fct.unl.pt (J.P.); e.owilla@campus.fct.unl.pt (E.C.); 2 Instituto de Telecomunicações, 1049-001 Lisbon, Portugal

**Keywords:** RF passive sensing, human posture classification, context awareness, machine learning, performance evaluation

## Abstract

This paper explores the opportunities and challenges for classifying human posture in indoor scenarios by analyzing the Frequency-Modulated (FM) radio broadcasting signal received at multiple locations. More specifically, we present a passive RF testbed operating in FM radio bands, which allows experimentation with innovative human posture classification techniques. After introducing the details of the proposed testbed, we describe a simple methodology to detect and classify human posture. The methodology includes a detailed study of feature engineering and the assumption of three traditional classification techniques. The implementation of the proposed methodology in software-defined radio devices allows an evaluation of the testbed’s capability to classify human posture in real time. The evaluation results presented in this paper confirm that the accuracy of the classification can be approximately 90%, showing the effectiveness of the proposed testbed and its potential to support the development of future innovative classification techniques by only sensing FM bands in a passive mode.

## 1. Introduction

Radio Frequency (RF) sensing systems can assess propagation environments and spectrum utilization to improve wireless systems’ performance [1]. RF sensing and context awareness systems were limited for specific purposes due to the technical challenges related to the understanding of most diversified contexts, where the techniques for classifying the scenario objects are more demanding [2]. The complexity of such challenges has motivated the adoption of more advanced techniques, including deep learning (DL) [3] and artificial intelligence (AI) techniques [4]. Several machine learning (ML) techniques have been used recently for RF human posture detection based on the RF Time of Flight (ToF) [5,6,7,8,9]. Although proposing different ML techniques, these works are based on active RF sensing systems.

Recently, significant research efforts have been carried out for detecting and classifying human activities (e.g., gestures, postures, gait, etc.) adopting passive RF sensing and DL/ML techniques. The work in [10] proposed a human body movement sensing system, operating in real time and focusing on detecting falling movement and locating more than one human body in the same area. The system exploits RF signals sent by radio devices that operate in the 2.4 GHz ISM band and detects events related to safety concerns by keeping track of radio signal strength indicator (RSSI) footprints. In [11], the influence of human movements on channel state information (CSI) was studied. Many experiments were carried out in order to extract features to detect human posture in complex scenarios, and commercial Wi-Fi devices were used to perform experiments that reached a detection rate of approximately 90%. An indoor motion recognition and classification method was proposed in [12] adopting a reference and a surveillance channel. Adaptive filtering was performed in order to eliminate the echoes from the surveillance signals. Then, a DL method was used to classify the time series signal into a certain motion, achieving an accuracy rate of approximately 70% in real-time operation. CSI was also considered in [13] to detect human presence using an RF phase and magnitude information to train DL models. In [14], a study was conducted on both passive and active RF sensing systems. Five different activities were distinguished, as well as their location within three indoor environments. In [15], a device-free system for fall detection was proposed, and CSI was exploited as the input to identify the activities in three different indoor scenarios using devices equipped with 802.11n cards.

Our work is motivated by the opportunities offered by the existing Frequency-Modulated (FM) radio broadcasting systems to detect indoor human motion through the analysis of the FM signal received by multiple devices. More specifically, our focus is on the development and testing of a passive RF testbed operating in FM radio bands, which allows experimentation with innovative human posture classification techniques. The contributions of this work include the following:

- The design of a testbed to perform passive RF sensing in FM radio bands. The testbed is prepared to not only gather RF information to be used in an offline manner but also to process the RF information in real time, allowing experimentation with innovative classification techniques;
- The specification of human posture scenarios that were used to sample RF information to be characterized in an offline manner to assist the development of the real-time sensing methodology;
- A general assessment of the importance of the multiple features computed from the received FM signals through the comparison of its score;
- A dynamic selection of the best features to adopt in the classification process according to the features’ score of the data gathered during an initial calibration scheme;
- The assessment of three classification techniques showing that simple classification schemes can easily achieve approximately 90% classification accuracy.

In our work, we are motivated by practical scenarios in which a person usually performs a task seated in a chair, such as train conductors or ship captains. The identification of these cases can support safety enforcement practices, as it is mandatory that some operations performed by the person in command must be carried out in a seated position and not in a standing position. The goal is to position the sensing system in front of a chair to identify if a person is in the standing position, seated in the chair, or not in front of the system. If nobody is identified in front of the sensing system, the state of the driving system must be switched to a nonoperational status. Otherwise, the driving system can be switched to an operational state, restricting the number of commands allowed when the driver is in the standing position and eliminating all restricted commands when seated. When compared with other systems, such as video recognition systems, the adoption of RF sensing systems for safety enforcement purposes does not raise privacy issues and is the main advantage of using it for the indicated purposes.

As far as we know, this is the first work focused on the design of passive RF systems for human posture classification operating in FM radio bands. The next section introduces the experimental setup. Section 3 describes the feature engineering methodology and introduces the classification steps compared in the testbed. Section 4 presents the performance results achieved with the classification methods run in the proposed testbed. Finally, Section 5 concludes this paper.

## 2. Experimental Testbed

This section describes the passive RF sensing system, the methodology followed to obtain the datasets, the scenarios to be classified, and the characterization of the datasets generated and used in this work.

### 2.1. RF Sensing System

The experimental setup used in this work consists of two similar Software-Defined Radio (SDR) devices and two identical directional antennas, two Low-Noise Amplifiers (LNAs), and computers to process the data. The SDR devices selected for this work were the *Nuand* bladeRF 2.0 micro xA4 SDR boards that offer 2 × 2 Multiple-Input Multiple-Output (MIMO) streaming, supporting the connection of 2 external receiving antennas (RX) and 2 external transmitting antennas (TX). The receiving RF chains of the SDRs are connected to a bias-tee, the *Nuand* BT-200 wideband LNA, to boost the received signal. The bladeRF’s complete specifications are presented in Table 1.

**Figure 1:**
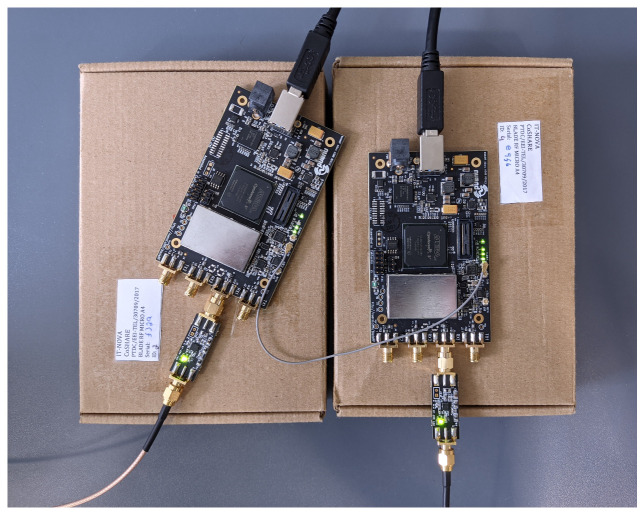
Details of the two bladeRF 2.0 micro xA4 boards with their clocks connected.

In this work, the FM signal is broadcast by an outdoor commercial FM radio station, and the testbed is mainly focused on the acquisition of the FM signals through two different RF chains implementing the reference and surveillance channels that substantiate passive radar systems. The reference antenna is mainly aligned to receive the outdoor FM signal, while the surveillance antenna focuses on the area where the classification occurs. To minimize possible sample losses due to USB communication rate degradation, we implemented each one of the two RX chains in a different SDR board. Consequently, during the sample acquisition process, the two bladeRF boards need to be synchronized. For this purpose, we used a synchronization cable, illustrated in Figure 1, which is used for synchronizing multiple bladeRF devices in a multidevice setup. Synchronization is crucial in applications where precise timing across multiple SDRs is necessary, such as in distributed MIMO (Multiple-Input Multiple-Output) systems or other advanced radio communication setups. The cable is used to connect specific synchronization ports on the bladeRF devices, allowing them to share timing information and maintain synchronization. This is important in scenarios where multiple devices need to operate together seamlessly, exchanging data without timing discrepancies. In our work, we used a master clock signal to synchronize the two *Nuand* bladeRF 2.0 micro xA4 boards.

This is performed by selecting the internal clock of one of the boards, say board 1, as the master clock. The master clock signal is then transmitted through the synchronization cable to board 2, which instead of using its internal clock will use the clock signal provided by board 1. Consequently, both boards use the same reference signal, and the samples gathered by each board are then synchronized. The boards allow this kind of operation by initially running a batch command that identifies which source clock is adopted by each card, and subsequently, they run a synchronization procedure that identifies the clock signal and regulates the synchronization parameters needed to successfully lock the synchronization loop. We connected the two SDR’s clocks using the synchronization cable, and we configured them accordingly. The port CLK_OUT from the device connected to the reference antenna is connected to the port CLK_IN of the SDR connected to the surveillance antenna, so the clock signal of the reference device serves as the master clock for both SDR boards.

**Figure 2:**
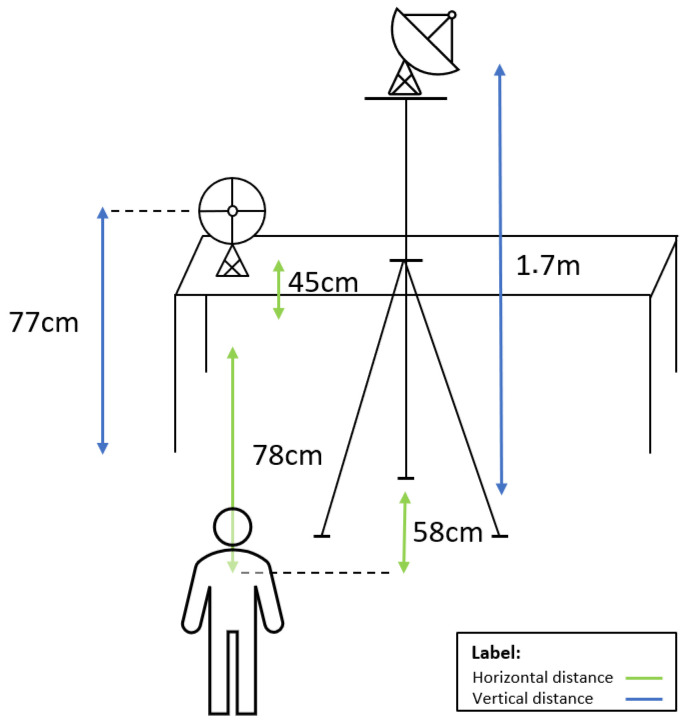
Schematic of the RF sensing system setup.

The schematic depicted in Figure 2 represents the passive RF sensing system from a person’s perspective in which relevant measurements are also provided. A surveillance antenna is pointing to the person whose posture we want to recognize (at a horizontal distance of 78 cm), and the reference antenna is pointing to the outdoor FM transmitting antenna. Additionally, the angle between the pointing direction of the reference antenna and the surveillance antenna is 90 degrees, making them approximately orthogonal to each other. We used two highly directional log-periodic antennas, the amateur radio design model WA5VJB, and their features are presented in Table 2.

### 2.2. Posture Classes

**Figure 3:**
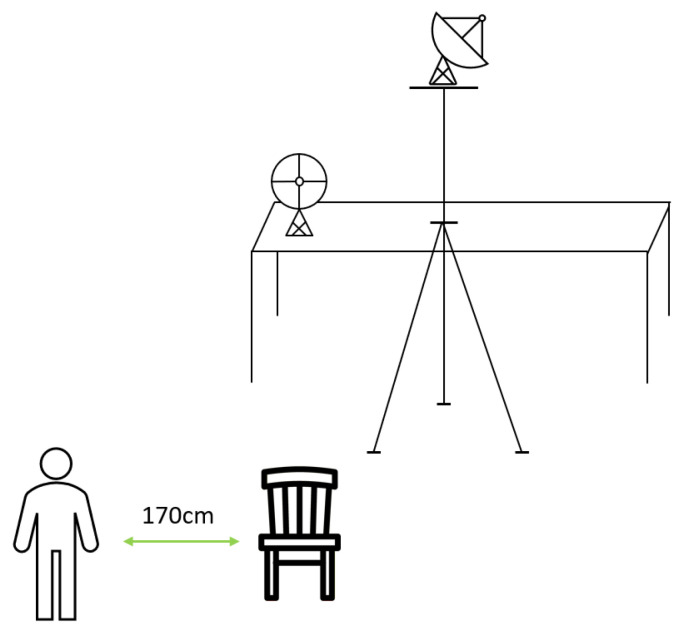
Schematic of a person performing the posture labeled as Class 2—None.

Aiming to work with realistic data acquired through the proposed testbed, we tested different human postures to be recognized in two different laboratory rooms (main lab and secondary lab). When collecting data from each laboratory, the setup layout and the distances between the sensing devices, illustrated in Figure 2, were kept as consistent as possible. This way, despite changing the environment, other variables were kept constant. The person whose posture we want to recognize performed three postures, described in Table 3, which are labeled in different classes. Class 0 represents a human posture where the person is standing in front of a chair and facing the surveillance antenna. In Class 1, the person is sitting on a chair and facing the surveillance antenna as well. As depicted in Figure 3, in the None class (Class 2) the person must be approximately 170 cm away from the chair without moving. Class 2 roughly represents the case when no human is presented in front of the RF system. Given the high directionality of the surveillance antenna, the power of the signal reflected by a human 170 cm away from the chair is significantly lower, as we experienced in the lab. For this purpose, we considered Class 2 to be representative of the case when no human is in the room, although a lower power is sensed when the human is effectively outside the room. This assumption was made to allow people to test the prototype without leaving the room.

### 2.3. Software

The synchronized SDRs connected to the reference and surveillance antennas are controlled through software using the GNU Radio development toolkit. GNU Radio [16] is a free and open-source software toolkit for studying, developing, and setting up SDR systems. Given that GNU Radio provides great flexibility when building a Graphical User Interface (GUI), this toolkit was chosen to program the SDR systems. We used the GUI GNU Radio Companion (GRC) to create flowgraphs of code blocks. GRC allows converting a flowgraph into a Python file, which can later be executed from the terminal. The necessary packages that allow the use of GNU Radio applications were installed with the aid of PyBOMBS [17].

**Figure 4:**
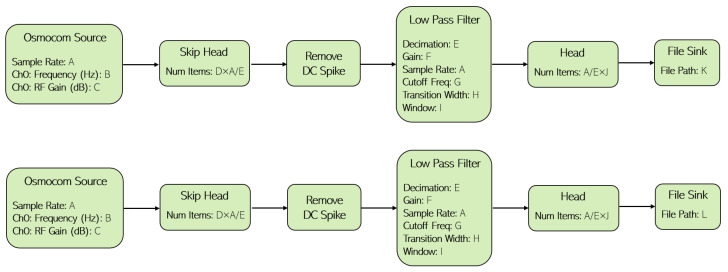
Block diagram of GNU Radio Companion flowgraph for offline data acquisition.

We acquired the data by running a Python script that is generated from the respective GRC flowgraph. A block diagram of the GRC flowgraph is illustrated in Figure 4. A description of the parameters set for the RF sensing system’s offline data acquisition is presented in Table 4.

The first two blocks depicted in Figure 4 (Osmocom Source) are the source blocks sampling the RF signal, which represent the two RX channels sampled by the two bladeRF 2.0 micro boards. For each one of the two sources, we tuned the center frequency to the radio station’s broadcast frequency B (in Table 4), we set the sample rate to A, and we also set the bias-tee gain to five different gains in C. The Analog-to-Digital Converter (ADC) collects the In-phase and Quadrature component (I/Q) samples from each RX channel, and these samples pass through the first two preprocessing blocks: the Skip Head and the Remove DC Spike blocks. The first one skips the first D×$\frac{A}{E}$ samples that come from the source block, representing the number of samples corresponding to D = 10 μs of data acquisition. This is an arbitrary value that was chosen to reflect some possible initial configurations from each bladeRF. The second block removes the center frequency I/Q DC spike with an Infinite Impulse Response (IIR) filter. In the next stage, the data are filtered by a Low-Pass Filter block. This block filters the receiving data with a Hamming window in order to select all frequencies lower than the defined cutoff frequency G and reject all higher frequencies. We set the transition width between stop-band and pass-band to H, the gain to F, and the decimation rate of the filter to E. This decimation rate redefines the sample rate to $\frac{A}{E}=\frac{4\mathrm{MS}/s}{16}=250$ kS/s. The purpose of this filter is to allow us to only work within the bandwidth of the baseband FM signal.

The Head block copies the first $\frac{A}{E}\times$J samples to the output. Note that, at this point, the sample rate is equal to 250 kS/s, and the Acquisition Period is the data acquisition time in which we used different times in J to form the datasets. Given the Acquisition Period, denoted by J, $\frac{A}{E}\times$J is equal to 5, 7.5, 22.5, and 45 MS, respectively, which represent the total amount of collected samples from each bladeRF 2.0 micro to for the offline datasets.

The last block of the diagram in Figure 4, File Sink, was used to write the samples stream to a binary file. The File Sink block generates binary files from the samples that come from the previous block. The generated binary file is composed of 8-byte I/Q pairs, meaning that the file is full of float32s in IQIQIQ order (i.e., 4 bytes for the real part of the complex number and 4 bytes for the imaginary part). Therefore, two binary files are generated, one for the reference antenna and another for the surveillance antenna, and their size, in bytes, can be determined by the expression $\frac{A}{E}\times J\times 4\times 2$.

The SDR implements the RF chain, the analog-to-digital conversion, and the digitalization and serialization of the sampled data through the USB link. However, the GNU Radio blocks described in Figure 4 and Table 4 run on the PC side, and only specific procedures may be executed in the SDR’s Field-Programmable Gate Array (FPGA), depending on the SDR’s firmware (e.g., Fast Fourier Transforms (FFT) and other signal processing procedures that can run faster when computed in the SDR’s FPGA).

### 2.4. Datasets

Initially, we acquired smaller datasets (lasting 20 and 30 s). Table 5 depicts a description of 13 different datasets. For each table row, only one of the two files generated by the antennas is described, since both files have equal sizes and formats. The first four datasets only target two of the three classes. Then, the next nine datasets already take into consideration all classes. We highlight that the last three datasets were also collected in the secondary lab, and its name holds the “_2” suffix, not to be confused with the datasets obtained in the same lab and with the same order of classes. Considering these smaller datasets, our objective was to understand if there was any visual difference between each class and if the order in which each class was collected by the system was relevant and influenced the results. The first datasets were also used to characterize the quality of different features computed from the data, and their performance was visually analyzed. To process and analyze each dataset, we read back the complex values stored in the generated binary files describing the complex samples acquired from the two antennas.

**Figure 5:**
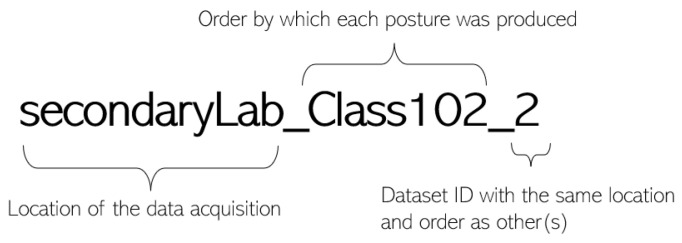
Structure of the name of each dataset.

The names of the datasets follow the structure depicted in Figure 5, where one of the datasets described above is used as an example.

Given that we did not consider transitions between the sampling process of each human posture, the data acquired between different postures need to be removed. Having a continuous flow of data being collected, we consider that the posture transitions do not last longer than 2 s and, consequently, we removed 2 s of signal at the beginning and at the end of each posture’s period acquisition. Table 6 provides information about the time division of each class for each dataset. Note that in this table, it is assumed that Class 0 is the first posture to be sampled, followed by Class 1, and finishing with Class 2.

After evaluating the smaller datasets, we acquired larger datasets lasting 90 and 180 s to have more data to work with and, consequently, have more representative data to analyze and make conclusions. Each dataset may be used to assess the real-time acquisition and evaluate the classification of human postures in an offline manner. Table 7 depicts a description of 30 different datasets. We collected the datasets on different days in order to take into consideration variations in the sampled signal. We also tested different bias-tee gains to analyze their impact.

We applied the same data processing in order to read the complex values from all of the datasets and, similarly, the transitions between postures were removed by applying the time division described in Table 8.

## 3. Feature Engineering and Classification

This section provides an initial solution to classify the human postures in the datasets. Our goal is to evaluate the capacity of the testbed to support experimentation in human posture recognition through a simple but effective classification methodology. We describe the roadmap followed to create, combine, and select the features computed from the acquired data that achieved the best performance for distinguishing the three classes of human postures. The classification methods are also described.

### 3.1. Classification Process Overview

The solutions proposed in this work consider that the classification algorithms take advantage of an initial stage of calibration, where the user is asked to pose according to each posture class, and labeled data are acquired for learning purposes. In the methodology, the datasets are split into calibration and test. The calibration data are used to extract knowledge that is further used to classify the test data. The calibration data are used to compute a set of features (presented in Section 3.2) and to identify a subset of features that better discriminate the calibration data. Note that different datasets, with different calibration data, can produce different feature selections. We also propose labeling the calibration data in order to follow a supervised learning approach in the classification process.

**Figure 6:**
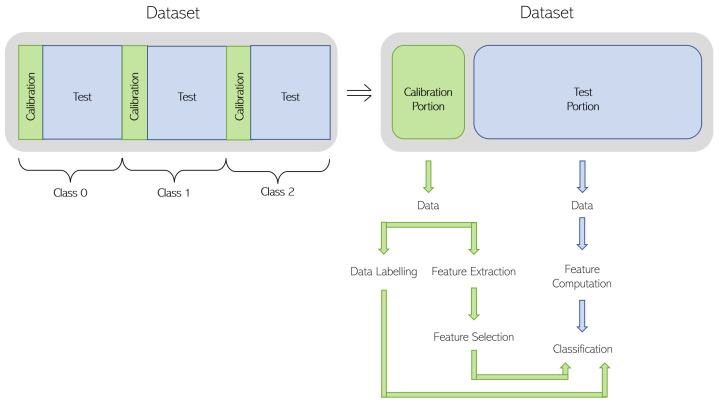
Block diagram with the approach followed to achieve the feature selection and classification goals.

An overview of the classification process is presented in the block diagram depicted in Figure 6, which summarizes the approach followed in this work. The top right dataset block is constructed by merging the calibration data (green color) from each class into the calibration portion and merging the test data (blue color) from each class into the test portion while maintaining the order of each class. As depicted, the calibration data are used to define which features should be computed and, in this way, the combination of the selected features is dynamically chosen. Then, the information regarding the selection of the features and the labeled data from the calibration portion are sent to the classification algorithm to predict the data in the test portion.

**Figure 7:**
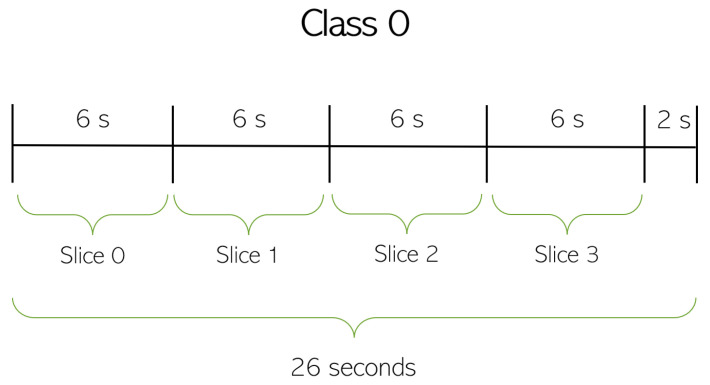
Time division of Class 0 into 6 s slices for a 90 s dataset.

The datasets lasting 90 s and 180 s are split into calibration and test portions. The calibration portion has a total duration of 18 s, which represents 6 s of data (or 1.5 MS) for each class. For a 90 s dataset, the test portion would have a total of 26×3−18 = 60 s of data (15 MS), and for each 180 s dataset, the test portion would have a total of 56×3−18 = 150 s of data (37.5 MS). Taking into consideration all the 90 s and 180 s datasets, different slices of 6 s can be chosen for each class of the calibration portion. To avoid the feature selection and classification steps being biased, different combinations of 6 s slices are considered for each of the three classes. The process is described in Figure 7 for datasets lasting 90 s. The Class 0 is divided into 4 slices of 6 s with 2 s remaining. The division is equal for every other class when considering 90 s datasets. Consequently, 4 different calibration portions are considered by randomly selecting one slice from each class until all the slices are used. Each calibration portion and sequence of slices is presented in Table 9. To give an example of how to read the information presented in Table 9 concerning the 90 s datasets, the first calibration portion is composed of slice 1 of Class 0, slice 0 of Class 1, and slice 1 of Class 2. It is also important to note that each calibration portion has a respective test portion, which corresponds to the missing data from the dataset when the calibration portion is removed.

With regard to a 180 s dataset, the division of each class follows the same reasoning depicted in Figure 7, where every class contains 9 slices of 6 s with 2 s remaining. For every 180 s dataset, 9 different calibration portions were considered by randomly selecting one slice from each class until all slices were used. Each calibration portion and sequence of slices are presented in Table 9 as well. Table 9 exemplifies how information is used. For datasets lasting 180 s, the first calibration portion is composed of slice 4 for Class 0, slice 8 for Class 1, and slice 2 for Class 2.

### 3.2. Feature Selection

To select the best features to represent the acquired data, we computed various features, and their performance was first visually analyzed considering all collected datasets. From the multiple features initially identified as hypothetical candidates, we selected 15 features of interest. The selection of the features was based on a quantitative benchmark (variance of the samples) to evaluate their capacity to discriminate the three classes in the datasets.

Let r be the matrix that contains the complex values collected by the reference antenna and s the matrix that contains the complex values collected by the surveillance antenna. Both r and s contain *N* columns and *M* rows, where *N* denotes the number of classes and *M* represents the number of samples per class. Table 10 identifies the 15 features adopted in the evaluation process. Note that when computing the Median Absolute Deviation (MAD), the central point used is the median.

Given the sample rate of 250 kS/s adopted in all datasets, we computed each feature for a sliding window of 25 kS. In each second of real-time acquisition, we were able to compute 10 feature outputs, which allowed 10 posture prediction outputs when neglecting the time required to compute each feature. The number of feature outputs for every dataset is described in Table 11.

To decrease the overall computation time of the classification process, only 2 of the 15 features were dynamically selected and used in the classification algorithm. It is important to highlight that some features are strongly correlated with others (e.g., Feature1 and Feature9); thus, the selection of such features would not add much information. First, we visually tested different combinations of two features with the datasets in Table 5 by performing a scatter of the two chosen features for each class.

The next step regarding feature selection relies on the identification of the two features that maximize the classification detection probability. In other words, the goal is to identify the two features that provide better separation between the sample clusters of the different classes. To evaluate the combinations of the features to be selected, we adopted the Analysis of Variance (ANOVA) method [18] for the calibration data from the 90 s and 180 s datasets. The ANOVA method is an efficient and simple feature selection technique that evaluates features through the variance between and within classes. The features achieving the highest F-statistic score can better discriminate the sampled data.

Taking into consideration the datasets lasting 90 s and 180 s, Table 12 and Table 13 present the mean, median, dissimilarity measure, and normalized variance of the 10 features obtaining the highest F-statistic values. The mean represents the average performance of a particular feature considering all datasets. The median, on the other hand, indicates the overall performance while ignoring the effect of outliers. The dissimilarity is computed as $\frac{\mathrm{Mean}-\mathrm{Median}}{\mathrm{Mean}}\times 100$ and represents the relative difference between the mean and the median value of a particular feature. The normalized variance is computed as $\frac{\mathrm{Variance}-\mathrm{Min}}{\mathrm{Max}-\mathrm{Min}}\times 100$, where Max is the maximum variance value from all 15 features and Min is the minimum variance value from all 15 features. The normalized variance indicates how distant the F-statistic values are from the mean value.

Each feature represented in Table 12 and Table 13 is color-labeled, and each color identifies which element was used to compute the feature. The blue color represents a feature computed from data acquired from the reference antenna, the yellow color represents a feature computed from data acquired from the surveillance antenna, and the orange color represents a feature that was computed using the data that resulted from the difference between the complex values acquired by the reference antenna (reference signal) and the complex values acquired by the surveillance antenna (surveillance signal).

Regarding the reference signal, Feature1 and Feature5 are the two features containing the lowest mean and median values. Comparing them, Feature5 exhibits lower mean and median values and higher dissimilarity and normalized variance values. For this reason, Feature1 is preferred. The features Feature9 and Feature14 are very similar in terms of their performance, both having a higher mean and median value than Feature1. Nonetheless, their dissimilarity and normalized variance values are significantly higher than Feature1. Given that a robust and consistent system is desired, Feature1 is preferred as a discriminant of the data obtained from the reference antenna.

Regarding the data obtained from the surveillance antenna, although Feature10 and Feature15 are the features that contain the highest mean value of all features, they also have the highest normalized variance values. Thus, Feature2 is preferred to describe the data obtained from the surveillance antenna.

Lastly, considering the features computed from the difference between the reference and surveillance data, Feature11 achieves the highest dissimilarity and normalized variance values of the three candidate features, and hence, it is discarded. The features Feature3 and Feature6 are very similar: Feature6 achieves a higher dissimilarity value and Feature3 has a higher normalized variance. Feature6 is chosen as the preferred one because we consider the variance as a more important metric in terms of measuring the overall performance of the system.

The two features to be used in the real-time classification process, out of Feature1, Feature2, and Feature6, are dynamically selected from the ones achieving the best ANOVA F-statistic.

### 3.3. Classification Methods

Regarding the classification methodologies, we exploited three different classification techniques:

- The sum of distances to all clusters’ points;
- Support Vector Machine (SVM);
- K-Nearest Neighbors (KNN).

In the first classification method, for each value pair obtained from the two selected features, we compute the total distance as the sum of all distances between that input and all points of the class cluster in the calibration dataset. Then, the method outputs the total distances for all clusters, and the cluster achieving the smallest total distance is considered as the predicted class. We used a total of 9 different distance metrics to compute this method: city block (or Manhattan), Euclidean, standardized Euclidean, squared Euclidean, cosine, Chebyshev (or infinite $L_{p}$ norm), Canberra, Bray–Curtis, and Mahalanobis.

Regarding the SVM classification technique, we used three different kernel types in the algorithm: linear, polynomial (3rd degree), and Radial Basis Function (RBF).

The KNN method is adopted using N=1,10,20,30,40,50,60 number of neighbors and, additionally, exploiting six distance metrics, namely city block, euclidean, cosine, Chebyshev, Canberra, and Bray–Curtis. For each distance metric, we computed the KNN for every different *N*.

For both the SVM and KNN classifiers, we used the *Scikit-learn* Python free software ML library [19] to train and classify the data.

**Figure 8:**
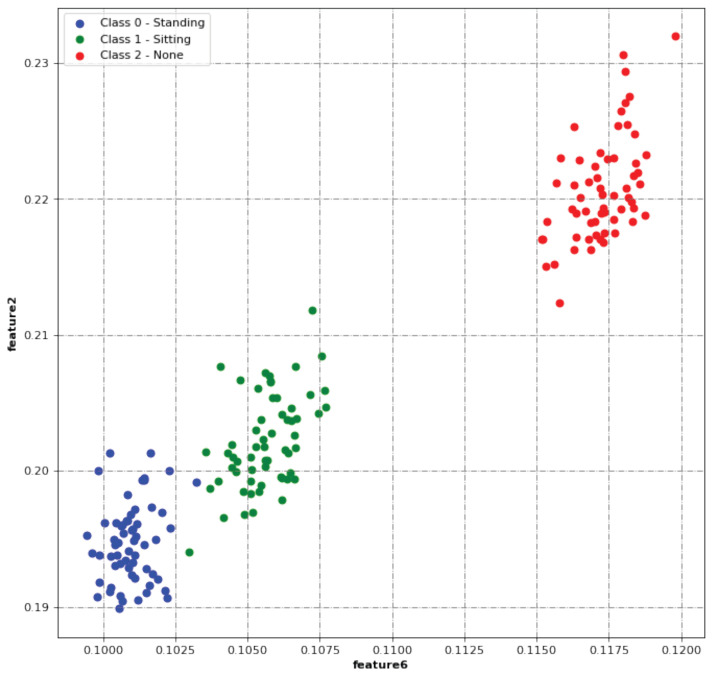
Classification results obtained with Feature6 and Feature2 (obtained with the secondaryLab_Class201 dataset).

Considering the offline classification step, only the 180 s datasets were used. This means that we used the data from 22 different datasets for this particular step. We performed an offline classification in which each classifier was trained with the labeled data received from the calibration data. The two selected features are computed for each 25 kS sliding window of each test portion. After computing the two features, a 2D feature output was constructed by placing the value of one feature on one axis and the value of the other feature on the other axis, as shown in Figure 8.

As described in Table 11, for a 180 s dataset, 560 feature outputs can be computed for each class. However, since only the test portions of each dataset are considered for the classification step, 560 − 60 = 500 feature outputs are computed for each class. Note that the 500 feature outputs are used to obtain 500 system classifications. Sample averaging was adopted, and 10 2D feature outputs were stored before feeding the classifier. This means that the classifier was fed with the 2D median of the accumulated feature outputs. Therefore, the total number of classifier predictions for each class can be summarized as described in Table 14.

## 4. Performance Evaluation

In this section, we assess the classification techniques described in Section 3.3 with the objective of detecting and classifying the three different human postures from the computed features.

The 180 s datasets were used in the classification and, consequently, in the performance evaluation results. Given that each dataset has nine different calibration portions (and the corresponding test portions), the accuracy of each class is defined as the mean accuracy considering all the calibration portions.

The expression $\frac{\mathrm{mean}\mathrm{accuracy}\mathrm{of}\mathrm{Class}0+\mathrm{mean}\mathrm{accuracy}\mathrm{of}\mathrm{Class}1+\mathrm{mean}\mathrm{accuracy}\mathrm{of}\mathrm{Class}2}{3}$ was computed to determine the accuracy of each classification method. KNN achieved the lowest accuracy, 61.030%, using the KNN classifier with K = 1 neighbors and using the cosine distance metric. On the other hand, SVM achieved the highest accuracy, 87.896%, with an RBF kernel. Considering each class individually, the mean accuracy was 87.434% for Class 0, 82.212% for Class 1, and 94.040% for Class 2. Taking into consideration the presented results, an SVM classifier with an RBF kernel was chosen for the classification tests.

The confusion matrix obtained with the SVM classifier with the RBF kernel was computed considering the data from all the test portions of all 180 s datasets and is represented in Table 15. The columns represent the class, and the rows represent the predicted class. As such, the diagonal matrix entries represent the cases where each class is correctly predicted. Computing the accuracy metric using the diagonal matrix entries will result in $\frac{8656+8139+9310}{(8656+1158+86)\times 3}\times 100=87.896\%$, which matches the value exposed above within the previous performance evaluation step. Note that the denominator operation corresponds to the total number of predictions.

Taking into consideration the confusion matrix, Table 16 presents the accuracy of the classification testbed. Notice that Class 2 achieves the highest accuracy values, followed by Class 0, and ending with Class 1. Taking into consideration the three classes, the system achieves a mean precision of 87.933%, a mean recall of 87.895%, a mean specificity of 93.948%, and a mean F1-score of 87.908%

As it was mentioned before, two out of three features are dynamically selected for each dataset. To evaluate the effectiveness of the selection of the features, we assess the frequency of each combination of the selected features for all 180 s datasets, as presented in Table 17. Although there is a combination of selected features with a significantly higher probability (Feature6 and Feature2), the proposed solution adapts to the different conditions imposed by the calibration data.

## 5. Conclusions

The main goal of this work was to design a system capable of detecting and classifying human postures based on passive RF sensing techniques. Firstly, an RF sensing solution based on two SDRs was described. The RF sensing system was used to collect data and generate datasets. These datasets were created taking into consideration three proposed classification scenarios (human postures) to be detected, which were labeled as classes. A features selection step was carried out applying an ANOVA feature selection algorithm. Regarding the classification stage, three different classification techniques were used: the sum of distances to all clusters’ points, SVM, and KNN. The SVM classifier with an RBF kernel achieved the highest performance, reaching an overall classification accuracy of 87.896%, a mean specificity of 93.948%, and a mean F1-score of 87.908%. The main conclusion of this work relies on the capacity of the proposed testbed to passively recognize human postures through FM radio bands, despite the simple classification algorithms proposed in this initial study. Future work includes the investigation and identification of different classification features and the evaluation of more adequate classification methods leveraged by the recent advances in machine learning and deep learning.

## Figures and Tables

**Table 1:** RF Specifications of the *Nuand* bladeRF 2.0 micro xA4.

RF Specifications	Min	Value	Max	Unit
RF Frequency Range (RX)	70	-	6000	MHz
RF Frequency Range (TX)	47	-	6000	MHz
ADC/DAC Sample Rate	0.521	-	61.44	MS/s
ADC/DAC Resolution	-	12	-	bits
RF Bandwidth Filter	<0.2		56	MHz
CW Output Power	-	+8	-	dBm

**Table 2:** Reference and surveillance antenna specification.

Feature	Value
Type	Periodic Log
Gain	6 dBi
Impedance	50 ohms
Frequency	850–6500 MHz
Dimensions	132 × 142 mm
Weight	33 g

**Table 3:** Human posture description and labeling.

Class	Posture Description
0	Standing in front of a chair
1	Sitting on a chair
2	None

**Table 4:** Description of the RF sensing system parameters set for offline data acquisition and described in the blocks in Figure 4.

Parameter Label	Value (s)
A	4 MS/s
B	104.3 MHz
C	16, 18, 20, 35, and 55 dB
D	10 μs
E	16
F	2 dB
G	100 kHz
H	10 kHz
I	Hamming
J	20, 30, 90, and 180 s
K	reference_file_path
L	surveillance_file_path

**Table 5:** Description of the 20 and 30 s datasets (main and secondary labs).

Dataset	Day	Bias-Tee Gain	Acquisition Period	Total Samples	File Size
mainLab_Class01	18/05	20 dB	20 s	5 MS	40 MB
mainLab_Class10	18/05	20 dB	20 s	5 MS	40 MB
mainLab_Class02	18/05	20 dB	20 s	5 MS	40 MB
mainLab_Class12	18/05	20 dB	20 s	5 MS	40 MB
mainLab_Class012	18/05	20 dB	30 s	7.5 MS	60 MB
mainLab_Class102	18/05	20 dB	30 s	7.5 MS	60 MB
mainLab_Class210	18/05	20 dB	30 s	7.5 MS	60 MB
secondaryLab_Class102	20/05	16 dB	30 s	7.5 MS	60 MB
secondaryLab_Class201	20/05	16 dB	30 s	7.5 MS	60 MB
secondaryLab_Class210	20/05	16 dB	30 s	7.5 MS	60 MB
secondaryLab_Class102_2	20/05	16 dB	30 s	7.5 MS	60 MB
secondaryLab_Class201_2	20/05	16 dB	30 s	7.5 MS	60 MB
secondaryLab_Class210_2	20/05	16 dB	30 s	7.5 MS	60 MB

**Table 6:** Time division of each class for all datasets lasting 20 s and 30 s.

Acquisition Period	Class 0	Class 1	Class 2	Class Duration	Class Samples
20 s	[2, 8] s	[12, 18] s	–	6 s	6×samplerate = 1.5 MS
30 s	[2, 8] s	[12, 18] s	[22, 28] s	6 s	6×samplerate = 1.5 MS

**Table 7:** Description of second datasets (main lab) that last 90 s and 180 s.

Dataset	Day	Bias-Tee Gain	Acquisition Period	Total Samples	File Size
mainLab_Class012_1	03/06	20 dB	90 s	22.5 MS	180 MB
mainLab_Class012_2	03/06	20 dB	180 s	45 MS	360 MB
mainLab_Class012_3	06/06	18 dB	180 s	45 MS	360 MB
mainLab_Class012_4	06/06	18 dB	180 s	45 MS	360 MB
mainLab_Class012_5	07/06	18 dB	180 s	45 MS	360 MB
mainLab_Class012_6	07/06	18 dB	180 s	45 MS	360 MB
mainLab_Class012_7	13/06	18 dB	180 s	45 MS	360 MB
mainLab_Class012_8	13/06	18 dB	180 s	45 MS	360 MB
mainLab_Class012_9	13/06	18 dB	180 s	45 MS	360 MB
mainLab_Class012_10	13/06	18 dB	180 s	45 MS	360 MB
mainLab_Class012_11	15/06	18 dB	180 s	45 MS	360 MB
mainLab_Class012_12	01/07	18 dB	180 s	45 MS	360 MB
mainLab_Class012_13	01/07	20 dB	180 s	45 MS	360 MB
mainLab_Class012_14	01/07	35 dB	180 s	45 MS	360 MB
mainLab_Class012_15	01/07	35 dB	180 s	45 MS	360 MB
mainLab_Class012_16	13/07	18 dB	90 s	22.5 MS	180 MB
mainLab_Class012_17	13/07	55 dB	90 s	22.5 MS	180 MB
mainLab_Class012_18	13/07	18 dB	90 s	22.5 MS	180 MB
mainLab_Class012_19	13/07	55 dB	90 s	22.5 MS	180 MB
mainLab_Class012_20	18/07	55 dB	180 s	45 MS	360 MB
mainLab_Class012_21	19/07	55 dB	180 s	45 MS	360 MB
mainLab_Class012_22	19/07	35 dB	180 s	45 MS	360 MB
mainLab_Class012_23	25/07	35 dB	180 s	45 MS	360 MB
mainLab_Class012_24	25/07	35 dB	90 s	22.5 MS	180 MB
mainLab_Class012_25	26/07	35 dB	180 s	45 MS	360 MB
mainLab_Class012_26	26/07	55 dB	180 s	45 MS	360 MB
mainLab_Class012_27	28/07	35 dB	180 s	45 MS	360 MB
mainLab_Class012_28	28/07	55 dB	90 s	22.5 MS	180 MB
mainLab_Class012_29	29/07	55 dB	180 s	45 MS	360 MB
mainLab_Class012_30	29/07	35 dB	90 s	22.5 MS	180 MB

**Table 8:** Time division of each class for the datasets lasting 90 s and 180 s.

Acquisition Period	Class 0	Class 1	Class 2	Class Duration	Class Samples
90 s	[2, 28] s	[32, 58] s	[62, 88] s	26 s	26×samplerate = 6.5 MS
180 s	[2, 58] s	[62, 118] s	[122, 178] s	56 s	56×samplerate = 14 MS

**Table 9:** Description of the sequence of the slices of the calibration portions for datasets lasting 90 s and 180 s.

	90 s Datasets	180 s Datasets
Class 0	[1, 2, 3, 0]	[4, 6, 5, 2, 0, 1, 7, 3, 8]
Class 1	[0, 1, 2, 3]	[8, 0, 4, 6, 1, 7, 2, 5, 3]
Class 2	[1, 0, 2, 3]	[2, 1, 4, 0, 7, 6, 5, 3, 8]

**Table 10:** Description of the 15 features adopted in this work (max denotes the maximum value, std represents the standard deviation, and skew denotes the skewness). Note that **r** (for reference signal) and **s** (for surveillance signal) are the matrices heretofore mentioned.

Feature Label	Formula
Feature1	max (∣**r**∣)
Feature2	max (∣**s**∣)
Feature3	max (∣**r**−**s**∣)
Feature4	std (∣**r**∣)
Feature5	std (∣**s**∣)
Feature6	std (∣**r**-**s**∣)
Feature7	skew (∣**r**∣)
Feature8	skew (∣**s**∣)
Feature9	mean (∣**r**∣)
Feature10	mean (∣**s**∣)
Feature11	mean (∣**r**-**s**∣)
Feature12	MAD (∣**r**∣)
Feature13	MAD (∣**s**∣)
Feature14	std (Re(**r**))
Feature15	std (Re(**s**))

**Table 11:** Description of the number of feature outputs for all datasets.

Acquisition Period	Class Samples	Number of Feature Outputs
20 s	1.5 MS	1.5 M/25 k = 60
30 s	1.5 MS	1.5 M/25 k = 60
90 s	6.5 MS	6.5 M/25 k = 260
180 s	14 MS	14 M/25 k = 560

**Table 12:** Features containing the best ANOVA F-statistic values of all the datasets lasting 90 s and 180 s.

	Feature1	Feature2	Feature3	Feature5	Feature6
Mean	661.292	2004.454	1812.913	513.850	1385.641
Median	1017.336	1519.037	1817.084	1046.483	1522.639
Dissimilarity	35.806%	72.440%	43.524%	32.851%	71.780%
Normalized Variance	0.334%	6.766%	3.195%	1.207%	2.807%
Key:					
	Reference		Surveillance		Difference

**Table 13:** Features containing the best ANOVA F-statistic values of all the datasets lasting 90 s and 180 s.

	Feature9	Feature10	Feature11	Feature14	Feature15
Mean	1584.780	5511.812	3217.418	1558.439	5395.585
Median	1017.336	1519.037	1817.084	1046.483	1522.639
Dissimilarity	35.806%	72.440%	43.524%	32.851%	71.780%
Normalized Variance	8.391%	100.000%	16.777%	7.470%	90.987%
Key:					
	Reference		Surveillance		Difference

**Table 14:** Description of the number of classifier predictions for each test portion.

	Number of Classifier Predictions
Class 0	500/10 = 50
Class 1	500/10 = 50
Class 2	500/10 = 50
Total	50 × 3 = 150

**Table 15:** Confusion matrix computed considering the data from all test portions of all 180 s datasets.

		True		
		Class 0	Class 1	Class 2
**Predicted**	Class 0	8656	1393	86
	Class 1	1158	8139	504
	Class 2	86	368	9310

**Table 16:** Accuracy of the performance evaluation metrics considering the data from the confusion matrix for all classes.

Class	Accuracy (%)	Precision (%)	Recall (%)	Specificity (%)	F1-Score (%)
Class 0	90.832	85.407	87.434	92.530	86.409
Class 1	88.475	83.043	82.212	91.606	82.625
Class 2	96.485	95.350	94.040	97.707	94.691

**Table 17:** Frequency of each combination of the selected features for all 180 s datasets.

	Number of Datasets	Percentage
Feature1 and Feature2	2	9.091%
Feature6 and Feature1	2	9.091%
Feature6 and Feature2	18	81.818%
Total	22	100%

## Data Availability

Data are contained within the article.
